# CHARACTERIZING GENERATIONAL CAREGIVING USING DATA FROM THE BRFSS

**DOI:** 10.1093/geroni/igac059.2046

**Published:** 2022-12-20

**Authors:** Benjamin Olivari, Nia Reed, Erin Bouldin, Eva Jackson, John Omura, Lisa McGuire, Janet Croft

**Affiliations:** CDC, Atlanta, Georgia, United States; CDC, Atlanta, Georgia, United States; University of Utah, Salt Lake City, Utah, United States; Alzheimer's Association, Chicago, Illinois, United States; CDC, Atlanta, Georgia, United States; CDC, Atlanta, Georgia, United States; CDC, Atlanta, Georgia, United States

## Abstract

As the proportion of family and friends in the U.S. providing informal care increases, it is important to understand how this may impact certain demographic groups. Millennials and older adults are two generational segments of caregivers of interest given the complexities associated with these groups. Millennials can be sandwiched between a growing older population of parents or grandparents while also raising young children, often providing care for both. Similarly, older adults frequently balance caring for spouses, themselves, and grandchildren. We characterized these two groups of caregivers using 2015-2019 Behavioral Risk Factor Surveillance System (BRFSS) data from the Caregiver Optional Module administered in 44 states, D.C., and Puerto Rico. Among 246,223 Module respondents, 21.3% reported providing care for a family member or friend. Among caregivers, 12.4% were identified as Millennials (aged 18-38 years), and 19.7% were older adults (aged≥65 years). About one-in-three Millennial caregivers reported providing care to a parent and half to another relative, such as a child or grandparent. While less than 4% of Millennial caregivers reported providing care for a spouse, nearly one-in-five older adult caregivers did. Although their care recipients may differ, both groups reported high intensity of care, with 30% of caregivers in each group providing 20 hours of care or more weekly. Caregiving among older adults can be further complicated by their own health difficulties, with over half of older adult caregivers reporting having two or more chronic conditions and one-third reporting a disability. BRFSS data may inform planning efforts pertaining to these caregiving groups.

